# Survival and Long-Term Cause-Specific Mortality Associated With Stage IA Lung Adenocarcinoma After Wedge Resection vs. Segmentectomy: A Population-Based Propensity Score Matching and Competing Risk Analysis

**DOI:** 10.3389/fonc.2019.00593

**Published:** 2019-07-03

**Authors:** Mengnan Zhao, Tao Lu, Yiwei Huang, Jiacheng Yin, Tian Jiang, Ming Li, Xinyu Yang, Cheng Zhan, Mingxiang Feng, Qun Wang

**Affiliations:** ^1^Department of Thoracic Surgery, Zhongshan Hospital, Fudan University, Shanghai, China; ^2^Eight-Year Program Clinical Medicine, Grade of 2014, Shanghai Medical College, Fudan University, Shanghai, China

**Keywords:** limited resection, lung adenocarcinoma, propensity score matching, competing risk analysis, SEER database

## Abstract

**Background:** Limited resection has been carried out increasingly in early stage NSCLC as an alternative to standard lobectomy. This study aimed to investigate the differences in survival and long-term cause-specific mortality between wedge resection and segmentectomy for treatment of stage IA lung adenocarcinoma.

**Method:** Cases with primary lung adenocarcinoma that received wedge resection and segmentectomy between 2004 and 2015 were selected from the Surveillance, Epidemiology, and End Results (SEER) database. Propensity score matching was performed to balance the baseline covariates. Long-term cause-specific mortality was investigated through competing risk analysis. The overall survival (OS) was estimated with the Kaplan-Meier method with the log-rank test. Univariate and multivariate Cox proportional hazards regression analyses were performed to identify the independent prognostic factors.

**Results:** Of the 3,046 cases included, 2,360 and 686 cases underwent wedge resection and segmentectomy, respectively. After propensity score matching, 686 pairs were selected. Segmentectomy was associated with a significantly better OS in stage IA2, grade I/II, female, and married patients. The segmentectomy group had a higher lung-cancer specific mortality in 65–74 years of age, stage IA1 and IA3, male, and married patients, and a lower chronic obstructive pulmonary disease (COPD) specific mortality in ≤64 and 65–74 years of age, stage IA1, IA2, and IA3, all grade, male, and married patients. The cardiovascular disease (CVD) specific mortality was also lower in the segmentectomy group in ≥75 years of age, stage IA1 and IA3, and grade I/II patients.

**Conclusion:** Wedge resection was inferior to segmentectomy in terms of OS regarding all included parameters. In most cases, the segmentectomy group had higher lung-cancer specific mortality and lower COPD and CVD specific mortality.

## Introduction

Lung cancer remains the leading cause of cancer-related deaths worldwide, and adenocarcinoma is the most frequent histological subtype (1, 2). Surgery is the cornerstone treatment for resectable tumors, which are found increasingly with the expanded use of low dose computed tomography (CT). Several treatment modalities have been applied to prolong the survival of patients with lung adenocarcinoma (3).

The selection of surgical procedure is largely based on the patient stage and general performance status. Lobectomy has been recommended as the standard procedure for early stage non-small-cell lung cancer (NSCLC) by the Lung Cancer Study Group (4). However, for patients with severely compromised pulmonary function, severe comorbidities, or other situations that preclude lobectomy, limited pulmonary resection (wedge resection and segmentectomy) is used as an effective alternative. In contrast to segmentectomy, wedge resection is generally considered a non-anatomical approach with less lymph node dissection (5).

A randomized controlled trial (RCT) reported by Ginsberg et al in 1995 has demonstrated that segmentectomy results in less risk of locoregional recurrence than wedge resection for NSCLC (4). Similarly, Dai et al. have reported that NSCLC patients with tumor sizes of 1–2 cm have a better survival outcome when treated with segmentectomy rather than wedge resection (6). Because most patients with stage I NSCLC are diagnosed at ≥ 65 years of age, the cause-specific mortality of such patients varies greatly at different times after diagnosis: elderly patients have a higher risk of chronic obstructive pulmonary disease (COPD), cardiovascular disease (CVD) and other diseases that could cause death independently of NSCLC (7). The identification of cause-specific mortality in different stratifications of resected early stage NSCLC would greatly aid in the selection of appropriate surgical approaches and surveillance after surgery. However, no large-scale retrospective or non-retrospective study has been conducted to compare the survival and long-term cause-specific mortality of wedge resection and segmentectomy in patients with stage IA lung adenocarcinoma. In this study, we retrospectively investigated the survival and mortality differences between wedge resection and segmentectomy for stage IA lung adenocarcinoma by performing propensity score matching and competing risk analysis using large data from the Surveillance, Epidemiology, and End Results (SEER) database. We hope that our results will help thoracic surgeons select the optimal surgical choice and personalized surveillance for patients with stage IA lung adenocarcinoma.

## Patients and Methods

### Ethics Statement

The approval for use of all the data was obtained through a request submitted to the SEER program. No approval by the institutional review board was sought, because SEER is a public database.

### Patients

Cases were collected from the SEER Program (www.seer.cancer.gov) SEER^*^Stat Database: Incidence—SEER 18 Regs Custom Data (with additional treatment fields), Nov 2017 Sub (1973–2015 varying) - Linked To County Attributes - Total U.S., 1969–2016 Counties, National Cancer Institute, DCCPS, Surveillance Research Program, released April 2018, based on the November 2017 submission.

**Figure 1:**
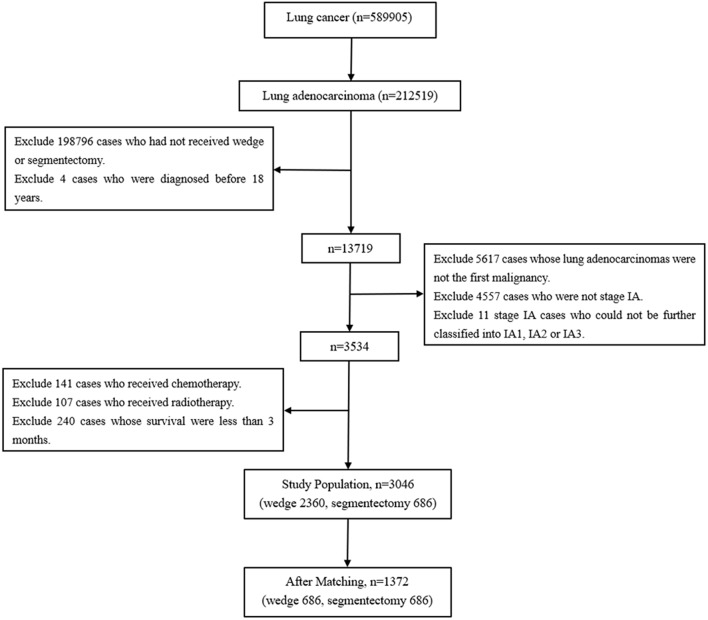
The flow diagram of the selection process for the study cohort.

Cases with tumors originating from the lung and diagnosed between 2004 and 2015 were selected by the variable “Primary Site – labeled.” Subsequently, cases with lung adenocarcinoma were identified on the basis of histology (International Classification of Diseases codes: 8140–8147, 8255, 8260, 8310, 8323, 8480, 8481, 8490, 8550, and 8572) (8). Cases that received wedge resection (SEER surgical code 21) and segmentectomy (SEER surgical code 22) were selected (9). Cases were excluded if they were diagnosed before the age of 18 years, were not stage IA, had received chemotherapy or radiotherapy, survived less than 3 months and had had a prior malignancy before the lung adenocarcinomas. The TNM-8 staging system was applied in this study (Figure 1) (10).

### Statistical Analysis

The cases were divided into two groups according to surgical mode (wedge resection vs. segmentectomy). Propensity score matching was used to lower the selection bias among the baseline variables in two groups, including age, sex, race, marital status, laterality, grade, TNM-8 stage, and surgical lobe (11, 12). Propensity scores were carried out by the “MatchIt” package in R version 3.4.4 (http://www.r-project.org/) (11, 13). Cases were matched on the basis of the propensity scores via the “nearest” method. The matching ratio was 1:1. The standardized difference of <10% was adopted to assess the balance of covariates before and after matching (14).

Correlations between two groups and different subgroups stratified by sex, laterality, race, marital status, year at diagnosis, state, surgical lobe and cause of death were analyzed with chi-square or Fisher's exact tests when appropriate. Because of the non-normal distribution of age, Wilcoxon rank-sum tests were performed to analyze their associations. Grade and stage were also analyzed with Wilcoxon rank-sum. The survival time was defined as the date of diagnosis to the date of death. Overall survival (OS) was estimated with the Kaplan-Meier method with the log-rank test for significance. Univariate and multivariate Cox proportional hazard regression analysis were performed to identify the independent prognostic factors. Related clinicopathological factors with *p* < 0.05 in univariate analyses were adjusted.

Causes of mortality were categorized as lung cancer; CVD, including diseases of the heart, hypertension without heart disease, atherosclerosis, aortic aneurysms and dissection, cerebrovascular diseases, and other diseases of the arteries, arterioles, and capillaries; COPD; and other diseases. The cumulative incidence of cause-specific mortality was calculated by using competing risk analysis.

All statistical analyses were performed in SPSS (version 24; IBM, Armonk, NY, USA). All tests were two-sided, and *p* < 0.05 were considered significant.

## Results

### Patient Characteristics

As shown in Figure 1, lung adenocarcinomas composed approximately 36.03% (212519/589905) of all lung cancer cases between 2004 and 2015. Cases diagnosed between 1973 and 2003 were all excluded because of a lack of TNM stage information. Among the entire the study population, 77.48% (2,360/3,046) and 22.52% (686/3,046) cases underwent wedge resection and segmentectomy, respectively. Patients with wedge resection were more likely to have tumors originating from the right lung (*p* = 0.006), to be diagnosed with early stage (*p* < 0.001), and to die of COPD/CVD (*p* = 0.002) (Table 1).

**Table 1:** Patient characteristics before and after propensity score matching.

**Characteristics**	**Before matching**				**After matching**			
	**Wedge**	**Segment**	***p-*value**	**SD (%)**	**Wedge**	**Segment**	***p-*value**	**SD (%)**
Total number	2360	686			686	686		
Age (years)	70 (25–91)	69 (37–91)	0.160	−5.309	69 (36–91)	69 (37–91)	0.251	−6.078
Removed lymph nodes	1 (0–90)	3 (0–54)	< 0.001^*^	35.745	1 (0–90)	3 (0–54)	< 0.001^*^	25.641
Sex			0.320				0.739	
Male	972	268		−4.325	262	268		1.796
Female	1388	418		4.325	424	418		−1.796
Laterality			0.006^*^				0.627	
Left	1000	331		11.828	340	331		−2.625
Right	1360	355		−11.828	346	355		2.625
Race			0.200				0.781	
White	2010	592		3.225	600	592		−3.455
Black	223	50		−7.808	49	50		0.563
Other	120	43		5.116	37	43		3.733
Unknown	7	1		−3.211	0	1		5.403
Marital status			0.180				0.880	
Married	1273	396		7.628	387	396		2.651
Unmarried	972	263		−5.822	272	263		−2.690
Unknown	115	27		−4.568	27	27		0
Year at diagnosis^a^			0.001^*^				0.126	
State^a^			< 0.001^*^				0.959	
Surgical lobe			< 0.001^*^				0.699	
Upper	1508	411		−8.215	425	411		−4.184
Middle	104	10		−17.547	10	10		0.000
Lower	719	262		16.327	250	262		3.617
Overlapping	6	0		−7.140	0	0		0
Unknown	23	3		−6.420	1	3		5.409
Grade			0.463				0.661	
I	649	179		−3.177	191	179		−3.942
II	1038	336		10.031	323	336		3.794
III	496	129		−5.542	131	129		−0.744
IV	15	4		−0.675	6	4		−3.428
Unknown	162	38		−5.496	35	38		1.949
Stage			< 0.001^*^				0.664	
IA1	535	111		−16.457	110	111		0.397
IA2	1342	393		0.857	385	393		2.354
IA3	483	182		14.340	191	182		−2.949
Cause of death			0.004^*^				0.002^*^	0.001
Alive	1567	501		14.475	448	501		16.789
Lung cancer	417	112		−3.576	118	112		−2.342
COPD	89	14		−10.315	36	14		−17.177
CVD	112	19		−10.405	30	19		−8.649
Other diseases	175	40		−6.374	54	40		−8.085

*Wedge, patients with wedge resection; Segment, patients with segmentectomy; SD, standardized difference; COPD, chronic obstructive pulmonary disease; CVD, cardiovascular disease. The variable of age is presented as median and range because it is not normal distribution*.

a *Detailed data of year at diagnosis and state are listed in Table S1*.

* *Indicates p < 0.05*.

**Figure 2:**
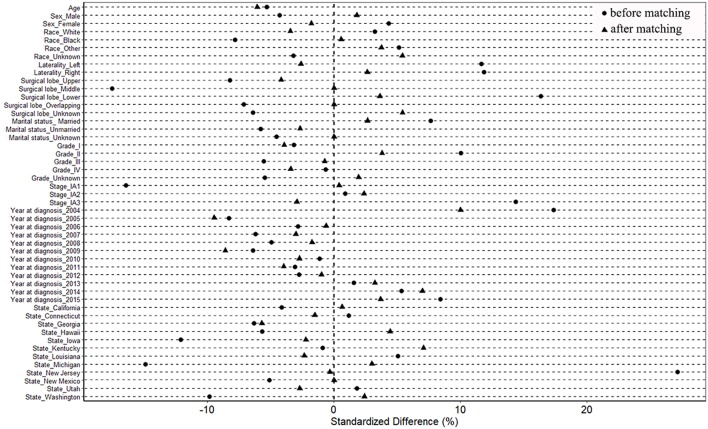
Standardized differences of baseline variables between patients with wedge resection and segmentectomy before and after propensity score matching.

After propensity score matching, 686 pairs were selected. All 686 patients with segmentectomy were included, and 686 of 2,360 patients with wedge resection were enrolled. There were no significant differences in the baseline variables between the two groups (Table 1 and Table S1). The absolute values of standardized differences in matched variables were all <10%, thus indicating that the variables were well balanced between the two groups after matching (Table 1 and Table S1). Figure 2 shows that the two groups had similar propensity score distributions after matching.

### Surgical Outcome Analysis

**Figure 3:**
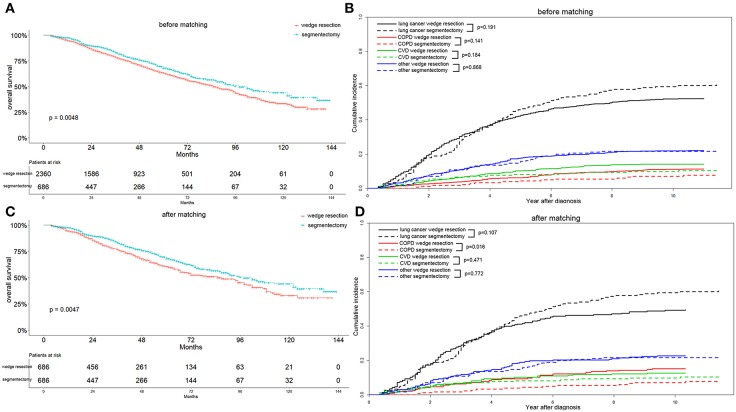
Overall survival **(A,C)** and cause-specific mortality **(B,D)** between patients with wedge resection and segmentectomy before and after propensity score matching.

Before matching, patients with segmentectomy had a significantly better OS (*p* = 0.005) than patients who underwent wedge resection (Figure 3A). The median OS values were 85 and 98 months for patients with wedge resection and segmentectomy, respectively. The 1-, 3-, and 5-year OS values were 95.0, 78.7, and 63.3% for the wedge resection group, and 97.5, 83.3, and 68.1% for the segmentectomy group, respectively. In the wedge resection group, the 1-, 3-, and 5-year cause-specific mortality consisted of lung cancer (2.8, 13.3, and 22.0%), COPD (0.5, 1.6, and 3.5%), CVD (0.6, 2.9, and 6.0%), and other specific mortality (1.1, 4.9, and 10.4%). In the segmentectomy group, the 1-, 3-, and 5-year cause-specific mortality consisted of lung cancer (1.4, 10.4, and 21.8%), COPD (0.0, 0.6, and 2.5%), CVD (0.6, 2.3, and 3.4%), and other specific mortality (0.5, 4.2, and 7.5%) (Figure 3B).

After elimination of the difference in covariates that might affect the OS and cause-specific mortality, through propensity score matching, we found that patients with segmentectomy exhibited a better OS (*p* = 0.005) and lower COPD mortality (*p* = 0.016) (Figures 3C,D). Because all 686 patients with segmentectomy before matching were enrolled after matching, the survival and mortality for the segmentectomy group were the same as those before matching. The median OS value was still 85 months for patients with wedge resection. The 1-, 3-, and 5-year OS values were 5.6, 23.2, and 39.4%, respectively, for the wedge resection group. In the wedge resection group, 1-, 3-, and 5-year cause-specific mortality consisted of lung cancer (2.9, 13.4, and 22.6%), COPD (0.6, 3.0, and 15.4%), CVD (1.1, 3.1, and 5.8%), and other specific mortality (1.1, 5.7, and 22.0%).

We further investigated the correlation between OS and other variables in the matched study population. As shown in Table 2 and Table S2, univariate analysis revealed that age (*p* < 0.001), sex (*p* < 0.001), grade (*p* < 0.001), marital status (*p* = 0.028), stage (*p* < 0.001), and surgery (*p* = 0.005) were significant prognostic factors for OS. After multivariate analysis, age (*p* < 0.001), sex (*p* = 0.001), marital status (*p* = 0.047), grade (*p* < 0.001), stage (*p* = 0.004), and surgery (*p* = 0.009) were independent prognostic factors of OS.

**Table 2:** Univariate and multivariate analysis of overall survival after matching.

**Characteristics**	**Univariate**			**Multivariate**		
	**HR**	**95%CI**	***p-*value**	**HR**	**95%CI**	***p-*value**
Age (years)	1.039	1.028–1.051	< 0.001^*^	1.039	1.028–1.051	< 0.001^*^
Sex			< 0.001^*^			0.001^*^
Male	Reference			Reference		
Female	0.671	0.554–0.813	< 0.001^*^	0.711	0.584–0.867	0.001^*^
Laterality			0.553			
Left	Reference					
Right	0.944	0.780–1.142	0.553			
Race			0.438			
White	Reference					
Black	1.128	0.811–1.570	0.473			
Other	0.685	0.409–1.149	0.151			
Unknown	0.002	0–791532416	0.947			
Marital status			0.028^*^			0.047^*^
Married	Reference			Reference		
Unmarried	1.254	1.033–1.522	0.022	1.264	1.036–1.543	0.021^*^
Unknown	0.725	0.385–1.366	0.320	0.830	0.439–1.568	0.565
Year at diagnosis^a^			0.179			
State^a^			0.173			
Surgical lobe			0.697			
Upper	Reference					
Middle	1.116	0.527–2.363	0.775			
Lower	0.894	0.729–1.096	0.280			
Unknown	1.239	0.307–4.994	0.763			
Surgery			0.005^*^			0.009^*^
Wedge	Reference			Reference		
Segmentectomy	0.759	0.626–0.920	0.005^*^	0.774	0.638–0.939	0.009^*^
Grade			< 0.001^*^			< 0.001^*^
I	Reference			Reference		
II	2.041	1.533–2.716	< 0.001^*^	1.874	1.405–2.500	< 0.001^*^
III	2.414	1.761–3.308	< 0.001^*^	2.448	1.776–3.375	< 0.001^*^
IV	2.188	0.686–6.981	0.186	2.234	0.696–7.166	0.177
Unknown	1.422	0.839–2.412	0.191	1.611	0.946–2.743	0.079
Stage			< 0.001^*^			0.004^*^
IA1	Reference					
IA2	1.738	1.247–2.423	0.001^*^	1.454	1.040–2.033	0.029^*^
IA3	2.316	1.639–3.272	< 0.001^*^	1.757	1.237–2.496	0.002^*^

*HR, hazard ratio; CI, confidence interval*.

a *Detailed data of year at diagnosis and state are listed in Table S2*.

* *Indicates p < 0.05*.

### Subgroup Analysis

**Figure 4:**
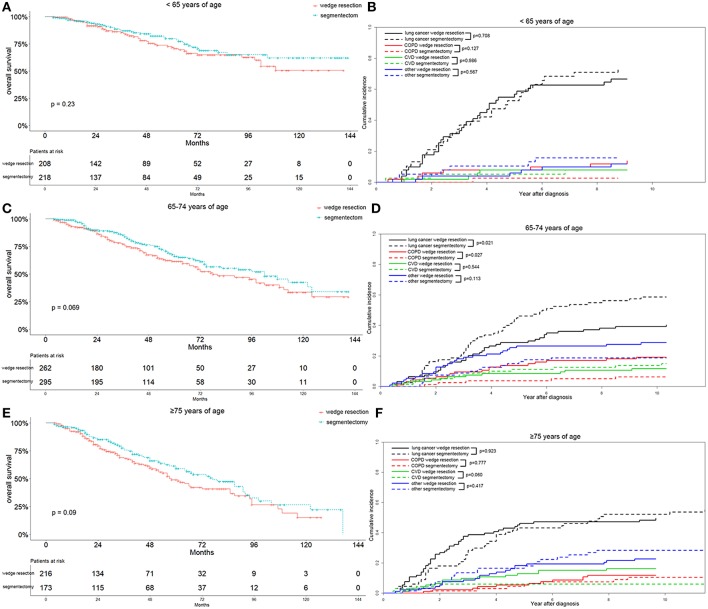
Overall survival and cause-specific mortality between patients with wedge resection and segmentectomy in ≤64 years of age **(A,B)**, 65–74 years of age **(C,D)**, and ≥75 years of age groups **(E,F)** after propensity score matching.

To better characterize the influence of surgical approaches on the survival and cause-specific mortality of patients with lung adenocarcinoma, we stratified the parameters of the matched patients on the basis of multivariable analysis. We observed that the segmentectomy group had a better OS, although the differences were not significant in patients ≤64 years of age (*p* = 0.230), 65–74 years of age (*p* = 0.069), and ≥75 years of age (*p* = 0.090) (Figures 4A,C,E). As for cause-specific mortality, the lung-cancer specific mortality was significantly higher in patients 65–74 years of age after segmentectomy (*p* = 0.021) (Figure 4D). In contrast, in patients ≥75 years of age, the lung-cancer specific mortality was higher in the wedge resection group before 7 years after diagnosis (Figure 4F). COPD specific mortality was significantly lower in the segmentectomy group in patients 65–74 years of age (*p* = 0.027), as was also observed in patients ≤64 years of age (Figures 4B,D). In patients ≥75 years of age, the COPD specific mortality was similar between the two groups (Figure 4F). Regarding CVD specific mortality, a lower mortality was observed in the segmentectomy group, only in patients ≥75 years of age (Figure 4F).

**Figure 5:**
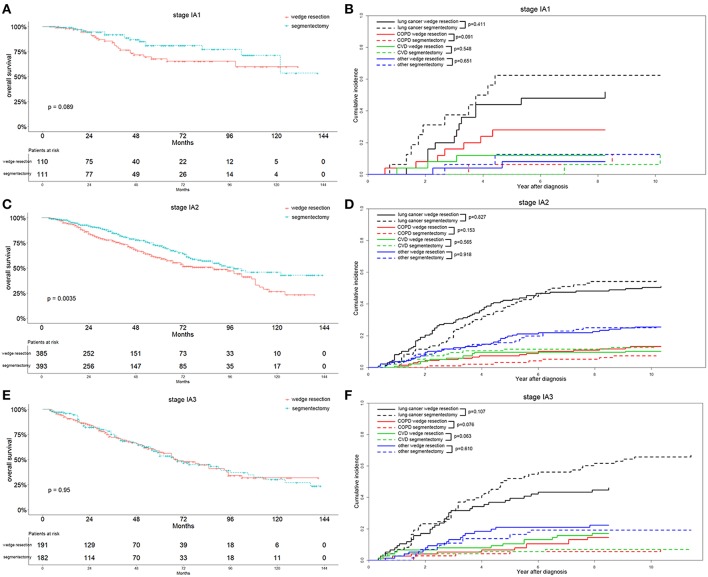
Overall survival and cause-specific mortality between patients with wedge resection and segmentectomy in stage IA1 **(A,B)**, stage IA2 **(C,D)**, and stage IA3 groups **(E,F)** after propensity score matching.

**Figure 6:**
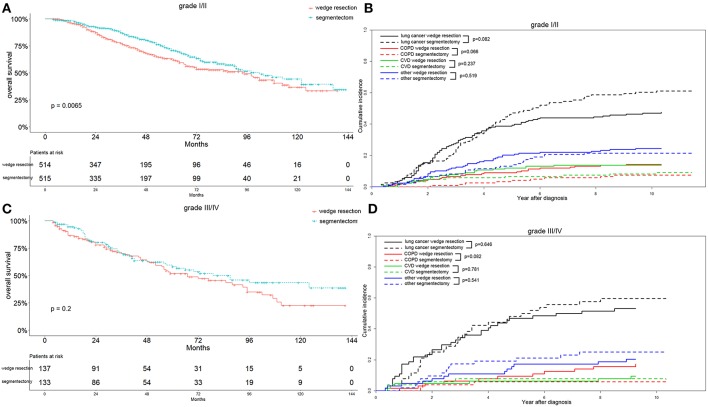
Overall survival and cause-specific mortality between patients with wedge resection and segmentectomy in grade I/II **(A,B)** and grade III/IV **(C,D)** groups after propensity score matching.

In the recent 8th TNM staging system, T1 classification of lung adenocarcinoma was divided into T1mi, T1a ( ≤ 1 cm), T1b (1-2 cm), and T1c ( ≤ 3 cm) (15). Analyzing the OS differences in each IA stage between two groups, we found that the OS was significantly better in the segmentectomy group in the IA2 stage (*p* = 0.004) (Figure 5C). The same association was also observed in the IA1 stage, although the result was not significant (*p* = 0.089) (Figure 5A). Nevertheless, the OS was similar between the two groups in IA3 stage (Figure 5E). Lung-cancer specific mortality was lower in the wedge resection group in stages IA1 and IA3, and was higher in the wedge resection group in stage IA2 before 6 years after diagnosis (Figures 5B,D,F). COPD specific mortality was higher in the wedge resection group in stages IA1, IA2, and IA3 (Figures 5B,D,F). CVD specific mortality was higher in the wedge resection group in stages IA1 and IA3, and was lower in in stage IA2 (Figures 5B,D,F). Regarding the differentiation grade, the segmentectomy group had a better OS for patients with well/moderately differentiated (grade I/II) tumors. The survival was comparable between the two groups with III/IV grade (Figures 6A,C). Patients with grade I/II and III/IV tumors exhibited a lower lung-cancer specific mortality in the wedge resection group after 4 years after diagnosis, and the COPD specific mortality was higher in the wedge resection group. The CVD specific mortality was also higher in the wedge resection group in grade I/II cases and was similar between the two groups in grade III/IV cases (Figures 6B,D).

**Figure 7:**
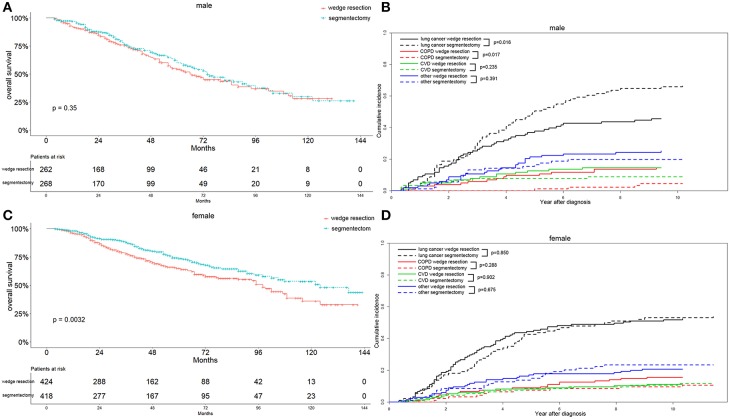
Overall survival and cause-specific mortality between patients with wedge resection and segmentectomy in male **(A,B)** and female **(C,D)** groups after propensity score matching.

**Figure 8:**
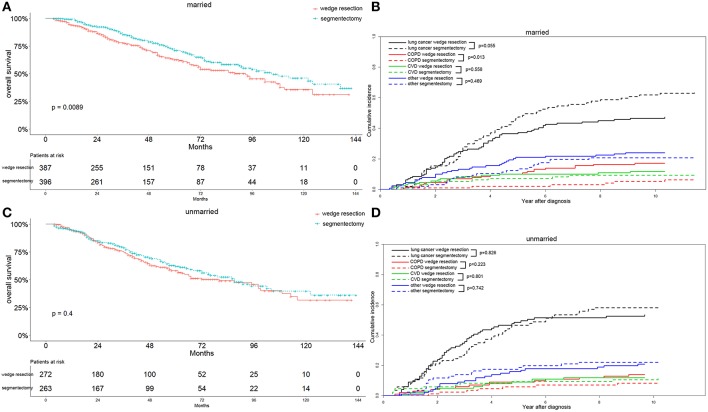
Overall survival and cause-specific mortality between patients with wedge resection and segmentectomy in married **(A,B)** and unmarried **(C,D)** groups after propensity score matching.

Interestingly, we observed that females with segmentectomy had a significantly better OS than those in the wedge resection group (*p* = 0.003), a result not observed in male patients (Figures 7A,C). Lung-cancer specific mortality was significantly higher in the segmentectomy group in male cases (*p* = 0.016) and comparable in female cases. However, the COPD specific mortality was significantly higher in the wedge resection group in male cases (*p* = 0.017), a result also observed in female cases. Both male and female cases had a similar CVD specific mortality between two groups (Figures 7B,D). Married patients also had a significantly better OS after receiving segmentectomy, and the results were similar between the two groups among unmarried patients (Figures 8A,C). Lung-cancer specific mortality was higher in the segmentectomy group in married cases and was similar between the two groups in unmarried cases. COPD specific mortality was significantly higher in wedge resection group in married cases (*p* = 0.013) and was comparable between the two groups in unmarried cases. CVD specific mortality was similar between the two groups in married and unmarried cases (Figures 8B,D).

In summary, the segmentectomy group showed a significantly better OS associated with stage IA2, grade I/II, female, and married patients. Patients with wedge resection did not show a better OS in relation to all parameters included in the study. Additionally, the segmentectomy group had higher lung-cancer specific mortality in 65–74 years of age, stage IA1 and IA3, male, and married patients, and a lower COPD specific mortality in ≤64 and 65–74 years of age, stage IA1, IA2, and IA3, all grade, male, and married patients. The CVD specific mortality was also lower in the segmentectomy group in ≥75 years of age, stage IA1 and IA3, and grade I/II patients.

## Discussion

In this study, we investigated the survival and long-term cause-specific mortality associated with wedge resection and segmentectomy in 3,046 patients with stage IA lung adenocarcinoma, by using propensity score matching to balance baseline covariates. We observed that wedge resection appeared to be inferior to segmentectomy in terms of OS regarding all included parameters. In most cases, the segmentectomy group had a higher lung-cancer specific mortality and lower COPD and CVD specific mortality. To the best of our knowledge, few studies about the survival and long-term cause-specific mortality in stage IA lung adenocarcinoma after wedge resection vs. segmentectomy had been published, especially with such a large number of cases.

Dziedzic et al. have reported that segmentectomy, compared to wedge resection, was associated with a significantly better OS in stage I NSCLC. However, the association was not significant after propensity score matching (16). As shown in Table 1, after matching, the significant differences in sex, chemotherapy, radiotherapy, laterality, surgical lobe, grade, and stage between two groups, which might markedly affect survival, were eliminated. Propensity score matching has played an increasingly important role in clinical analysis (12, 16, 17). As a statistical tool, it can generate a proper randomization in case selection in retrospective studies to reduce selection bias (18).

Competing risk analysis has been used frequently in the analysis of survival data. The primary event of interest is often precluded by a competing event; for instance, if the primary event of a study is death from lung cancer, death due to non-lung cancer diseases is a competing risk. In such circumstances, the lung cancer specific survival will be overestimated in the presence of competing events, and this overestimation can be reduced through competing risk analysis (19). Because most NSCLC patients are diagnosed at older ages, mutually competing diseases associated with elderly status become increasingly important in the cause of death (7). Thus, it is crucial to correctly analyze cause-specific mortality in the presence of other competing causes by using competing risk analysis, which can provide a reference for personalized therapy, follow-up, and surveillance.

Smith et al. have observed that in patients ≤70 years of age, the OS is better after segmentectomy compared with wedge resection for stage I NSCLC. The survival advantages of segmentectomy were not found to be significant in patients >70 years of age (9). In our study, after detailed grouping of age, we observed that segmentectomy was associated with better OS in the three age groups, but the results were not significant, possibly because of the different statistical methods used (Cox regression analysis in the previous study and the Kaplan-Meier method with log-rank test in our study). In the segmentectomy group, the lung cancer specific mortality was comparable in patients ≤64 years of age, significantly higher in patients of 65–74 years of age, and lower at first in patients ≥75 years of age. The COPD specific mortality was lower in patients ≤64 and 65–74 years of age and similar in patients ≥75 years of age. However, the CVD specific mortality was similar in patients ≤64 and 65–74 years of age and lower in patients ≥75 years of age in the segmentectomy group. The reasons for these results may be that, as age increased, chronic diseases, such as COPD and CVD, occurred with increasing severity. Moreover, patients who received segmentectomy were meant to have a better performance status.

In agreement with our research, a previous study has also found that females are more likely to develop lung adenocarcinoma, thus making the selection of optimal surgical procedures in females more meaningful (20). Our study indicated that females showed a significant survival benefit after segmentectomy. Regarding males with stage IA lung adenocarcinoma, there was no significant OS difference between the two groups, and the survival curves almost overlapped. Nevertheless, male patients in the segmentectomy group showed a significantly higher lung cancer specific and a lower COPD mortality, whereas female patients showed similar mortality between the two groups. To date, no research on similar phenomena has been published; therefore, further research is necessary.

Interestingly, we observed that married patients had a better OS than those who were unmarried after limited resections. Wu et al. also reported that married patients exhibited a better OS after surgery in stage I NSCLC (21). They thought this may be the result of better compliance and less emotional burden in married patients.

Traditionally, lobectomy is performed as the standard surgical procedure for stage I ( ≤ 3 cm) NSCLC. However, for patients who cannot tolerate lobectomy, limited resection (wedge resection and) is used as an alternative (6). Additionally, patients after limited resection are more tolerant to future lung resections if further lung cancers occur (22). In segmentectomy, compared with lobectomy, whether the extent of resection of limited resections is sufficient for early stage NSCLC remains controversial. Malignant cells infiltrate along the bronchial vascular bundles and lymphatic vessels to the hilum of the lung. Even small lung cancers have the potential to spread locally or systemically (23). Kates et al. have observed that lobectomy does not result in a significantly better OS or lung-cancer-specific survival rates than limited resection for stage I NSCLC <1 cm in size, before and after balancing of all covariates (24). Okada et al. have also demonstrated that in ≤2 cm stage I NSCLC, limited resection and lobectomy have similar curability, incidence of postoperative complication, and local recurrence. Moreover, the two groups had similar disease-free and OS (25).

Wedge resection is less technically challenging and may be preferable to segmentectomy in the treatment of early stage NSCLC. Because of the non-anatomic and lesser extent of lymph node dissection, its curative effects have been questioned. The only RCT comparing lobectomy vs. limited resection in early stage NSCLC was reported by Ginsberg et al in 1995; that study revealed that wedge resection results in a higher risk of local recurrence than segmentectomy (4). Sienel et al. have also reported that wedge resection, compared to segmentectomy, was associated with a significantly increased local recurrence in stage IA (both ≤3 cm and ≤2 cm) NSCLC. However, the distant metastasis was similar between the two groups. Furthermore, the segmentectomy group showed a better cancer-related 5-year survival in stage IA (both ≤3 cm and ≤2 cm) NSCLC (22). The eighth TNM stage of lung cancer divides the T1 classification into T1mi, T1a ( ≤ 1 cm), T1b (1–2 cm), and T1c ( ≤ 3 cm) for significantly different 5-year survival (15). In agreement with our findings, Dai et al have also demonstrated that wedge resection leads to poorer OS and lung cancer-specific survival in stage IA2 (1–2 cm) NSCLC than segmentectomy, whereas they found that the survival did not differ between the two groups in stage IA1 (<1 cm) NSCLC, findings also based on the SEER database. Nevertheless, all cause specific mortality did not prominently differ between the two groups in stage IA1, IA2, and IA3. We believe that stage IA3 NSCLC is more invasive than stage IA1 and IA2. Thus, the segmentectomy group showed comparable survival to that of the wedge resection group in stage IA3 NSCLC.

This study has several limitations that should be noted. First, micropapillary-predominant and solid with mucin-predominant lung adenocarcinomas showed better survival than adenocarcinoma *in situ*, minimally invasive adenocarcinoma, and lipidic-predominant adenocarcinoma (26). However, the SEER database does not provide information about histological subtypes. Therefore, we were unable to analyze the effects of different histological subtypes on patient survival and mortality after wedge resection or segmentectomy. Second, because targeted therapy and immunotherapy for lung adenocarcinoma have increasingly been applied, the survival of these patients may greatly differ from those without targeted therapy. We also did not analyze the effects of different targeted therapies and immunotherapy on survival and cause specific mortality in patients with wedge resection or segmentectomy, because of a lack of detailed data in the SEER database. Nevertheless, for early stage lung adenocarcinoma, the patients were less likely to receive such therapy; therefore, our results may not have been substantially affected. Third, our study was retrospective, and some bias was inevitable. Our results maybe weak and prospective studies with a larger randomized study cohort are needed to further validate our results. Finally, no data about the surgeon experience, volume and surgery approach (open vs. VATS) was available from the SEER database. And further researches are needed.

In conclusion, segmentectomy exhibited a better OS than wedge resection in stage IA lung adenocarcinoma. The segmentectomy group had a higher lung-cancer specific mortality, a lower COPD specific mortality and CVD specific mortality in most patients. And large-scale prospective studies are necessary to further validate our results.

## Author Contributions

MZ and TL: study design, analysis and interpretation of data, drafting the article, and final approval. YH: acquisition of data, drafting the article, revising the article, and final approval. JY, TJ, ML, and XY: analysis and interpretation of data, drafting the article, and final approval. CZ study design, analysis, and interpretation of data, drafting the article, revising the article critically for important intellectual content, final approval, agreement to be accountable for all aspects of the work. MF: study design, drafting the article, revising the article critically for important intellectual content, final approval, agreement to be accountable for all aspects of the work. QW: study design, drafting the article, revising the article critically for important intellectual content, final approval.

### Conflict of Interest Statement

The authors declare that the research was conducted in the absence of any commercial or financial relationships that could be construed as a potential conflict of interest.
