# Supplementary material for: Survival and Long-Term Cause-Specific Mortality Associated With Stage IA Lung Adenocarcinoma After Wedge Resection vs. Segmentectomy: A Population-Based Propensity Score Matching and Competing Risk Analysis
Source: Front Oncol. 2019 Jul 3;9:593. doi: 10.3389/fonc.2019.00593 (PMC6616069; doi:10.3389/fonc.2019.00593)
Supplement: Supplementary file 1 [file Table_1.docx]

**Table S1.** Year at diagnosis and state before and after propensity score matching.

| Characteristics | Before matching | | | |  | After matching | | | |
| --- | --- | --- | --- | --- | --- | --- | --- | --- | --- |
|  | wedge | segment | *p* value | SD (%) |  | wedge | segment | *P* value | SD (%) |
| Total number | 2360 | 686 |  |  |  | 686 | 686 |  |  |
| Year at diagnosis |  |  | 0.001* |  |  |  |  | 0.126 |  |
| 2004  2005  2006  2007  2008  2009  2010  2011  2012  2013  2014  2015 | 95  139  143  174  185  202  179  231  236  267  288  221 | 56  28  37  40  45  47  50  61  63  81  96  82 |  | 17.362  -8.315  -2.866  -6.213  -4.951  -6.408  -1.129  -3.079  -2.772  1.545  5.310  8.397 |  | 31  44  38  45  48  63  55  69  65  74  80  74 | 56  28  37  40  45  47  50  61  63  81  96  82 |  | 9.996  -9.474  -0.641  -3.024  -1.740  -8.597  -2.742  -3.983  -1.002  3.224  6.979  3.674 |
| State |  |  | <0.001* |  |  |  |  | 0.959 |  |
| California  Connecticut  Georgia  Hawaii  Iowa  Kentucky  Louisiana  Michigan  New Jersey  New Mexico  Utah  Washington | 782  202  273  26  83  181  59  183  396  20  20  135 | 214  61  66  4  11  51  23  29  192  3  7  25 |  | -4.155  1.179  -6.329  -5.677  -12.137  -0.890  5.061  -14.902  27.138  -5.135  1.798  -9.839 |  | 212  64  78  2  13  39  26  25  193  3  9  22 | 214  61  66  4  11  51  23  29  192  3  7  25 |  | 0.630  -1.520  -5.710  4.419  -2.224  7.070  -2.357  2.999  -0.324  0.000  -2.716  2.405 |

Wedge: patients with wedge resection; Segment: patients with segmentectomy. SD: standardized difference.

* indicates *p* value < 0.05.
