# Supplementary material for: Survival and Long-Term Cause-Specific Mortality Associated With Stage IA Lung Adenocarcinoma After Wedge Resection vs. Segmentectomy: A Population-Based Propensity Score Matching and Competing Risk Analysis
Source: Front Oncol. 2019 Jul 3;9:593. doi: 10.3389/fonc.2019.00593 (PMC6616069; doi:10.3389/fonc.2019.00593)
Supplement: Supplementary file 2 [file Table_2.docx]

**Table S2.** Univariate analysis of overall survival after matching.

| Characteristics | Univariate | | |
| --- | --- | --- | --- |
|  | HR | 95%CI | *p* value |
| Year at diagnosis |  |  | 0.179 |
| 2004 | Reference |  |  |
| 2005 | 0.964 | 0.657-1.415 | 0.853 |
| 2006n | 0.999 | 0.685-1.458 | 0.996 |
| 2007 | 1.062 | 0.727-1.551 | 0.756 |
| 2008 | 0.850 | 0.572-1.263 | 0.422 |
| 2009 | 0.728 | 0.487-1.089 | 0.123 |
| 2010 | 0.724 | 0.473-1.108 | 0.137 |
| 2011 | 0.686 | 0.447-1.053 | 0.085 |
| 2012 | 0.658 | 0.405-1.068 | 0.090 |
| 2013 | 0.522 | 0.294-0.927 | 0.026* |
| 2014 | 0.638 | 0.325-1.253 | 0.192 |
| 2015 | 0.238 | 0.032-1.770 | 0.161 |
| State |  |  | 0.173 |
| California | Reference |  |  |
| Connecticut | 0.892 | 0.602-1.321 | 0.568 |
| Georgia | 0.905 | 0.646-1.269 | 0.563 |
| Hawaii | 1.809 | 0.668-4.897 | 0.243 |
| Iowa | 0.637 | 0.281-1.446 | 0.281 |
| Kentucky | 1.490 | 1.029-2.159 | 0.035* |
| Louisiana | 1.167 | 0.712-1.913 | 0.541 |
| Michigan | 0.856 | 0.522-1.404 | 0.538 |
| New Jersey | 0.952 | 0.743-1.220 | 0.698 |
| New Mexico | 1.618 | 0.400-6.547 | 0.500 |
| Utah | 0.368 | 0.091-1.487 | 0.161 |
| Washington | 0.589 | 0.299-1.158 | 0.125 |

HR: hazard ratio; CI: confidence interval.

**^＊^** indicates *p* < 0.05.
